# The Access to Grammatical Number in Spanish Children and Adults

**DOI:** 10.1007/s10936-023-10008-x

**Published:** 2023-09-01

**Authors:** Alberto Dominguez, Anthea Santos, Yang Fu

**Affiliations:** Instituto Universitario de Neurociencia (IUNE), Universidad de La Laguna, 38205 Tenerife, Spain

**Keywords:** Grammatical number, Lexical decision, Plural processing, Number acquisition, Dominant frequency

## Abstract

In Spanish, the plural form in plural dominant frequency pairs, like “diente/dientes” [tooth/teeth], occurs more frequently than the corresponding singular form. On the other hand, for the singular dominant frequency pairs such as “cometa/cometas” [kite/kites], the singular form is more common than the plural. The recognition of singular forms by adult readers is dependent on the dominance factor, while the identification of plural forms relies on the frequency of the stem. Given that age and reading experience may influence morphological processing of words, we investigate the representation of singulars and plurals in Spanish primary school children in Third Grade (8/9) and Sixth Grade (11/12) and adults through a lexical decision task. Though children’s lexical decisions were twice as slow as adults, the pattern of morphological processing was consistent across ages: dominant plural forms resulted in decision times that were comparable to those of non-dominant singular forms, while recognition of singular-dominant forms was quicker than recognition of plural non-dominant forms. It appears that singulars are accessed and stored in the lexical memory as separate entities, while plurals depend on their morphological closer relatives, in this case, the singular forms.

## Introduction

After 30 years of research on the processing of morphological number, the recognition of plurals is a topic in word recognition research that yields seemingly inconsistent results. A usual methodology to investigate how morphologically complex words are accessed and represented is the lexical decision task on the dominant and non-dominant singular and plural nouns. Pairs of words such as “diente/dientes” [tooth/teeth], in which the plural form is much more frequent than the singular one, are called plural dominants. On the contrary, pairs of singular dominant frequency words are those, such as “cometa/cometas” [kite/kites], in which the singular form is higher in frequency than the corresponding plural form. A well-established interpretation suggests that the correlation between dominance and reaction times indicates independent access and representation of singular and plural forms. On the contrary, if the plural access depends on the singular forms, no reaction times advantage of plural dominant forms over the corresponding singular forms is expected.

The classical Dual Route (race) Model by Schreuder and Baayen (1995) accounts for two routes in the process of singular and plural forms: a direct route which allows to accede and represent the whole word, and a decomposition route through which stem and affixes are identified. The velocity of both routes depends on several factors, among them, the frequency of the specific form, plural or singular, and the possibility to segment the word, not always possible, because Spanish, English or French, marks plural with a –s but they do not mark the singular, reason why it is not necessary to segment the singular. This asymmetry could be the reason for different recognition times for singular and plural forms of singular-dominant pairs “cometa/cometas” [kite/kites] versus plural-dominant pairs “diente/dientes” [tooth/teeth]. The dual route model would predict that singular forms of a singular dominant pair depend on their own frequency whereas plural forms could depend on the frequency of their corresponding singular. On the other side, the recognition of singular and plural forms of plural dominant items will not differ because the singular, e.g. “diente” [tooth] is accessed directly, employing long reaction times due to their low frequency. Plural recognition “dientes” [teeth] do not benefit on their higher frequency because their entry is done through a singular low-frequency form.

These are the results obtained in visual word recognition tasks in Spanish (Dominguez et al., 1999). The superficial mark “-s” in “diente-s” [teeth]) seems to be the information used by readers to recognize the plural of a noun, but the recognition of the complete word demands the identification of a stem, which corresponds to the singular form. Similar results had been obtained also in German (Baayen et al., 1997a) English (Sereno & Jongman, 1997) French (New et al., 2004), Italian (Baayen et al., 1997b) and Dutch (Reifegerste et al., 2017) and not only in comprehension but also in production tasks (Beyersmann et al., 2015). These consistent effects would be supporting an access to the entry of plurals across the singular corresponding form, because in Spanish, English, French, Italian, Dutch or German languages the plural marks are added to the singular form.

This panorama becomes complicated by the results of Gimenes et al. (2016) in a mega-study making an analysis of regression on the lexical decision times of 2954 French words, 1475 English words and 544 German words. A prediction of the authors, later confirmed by data, was that “…for the three languages, as plurals should activate singulars whenever they are processed, base frequency should be the only predictor for singulars (no effect of surface frequency)” (p. 318). The regression data obtained by the authors showed that times for singulars were better predicted by the frequency of the base than by its surface frequency, because singulars are, in fact, the base of the corresponding plural forms, “diente” [tooth] is a part of “dientes” [teeth]. Reaction times corresponding to the plural words, on the contrary, were well predicted by the base frequency as for the surface frequency. At first glance, these regression results appear incompatible with the dominance paradigm data, because Gimenes et al. would predict that singulars dominant and singulars non-dominant would obtain similar reaction times but, actually, singulars dominant spend less time than singulars non-dominant to be recognized. However, these results can hardly be compared with those derived from the classical model, since no direct comparison of singular and plural forms of the same stem was carried out.

On the other hand, these results seem congruent with the probabilistic point of view of Baayen et al., (2007, 2011), who consider two types of information to be taken into account by the linguistic processor to recognize compound words. *Paradigmatic* information basically refers to the number of words using a given constituent (e.g. a morpheme). It refers to the well-known effect of morphological family size. S*yntagmatic* information, on the other side, refers to the probability that a series of letters is followed by another series in different words; for example, the probability that a final -s determines the plural of a word. In both cases, the meaning attributed to the word can be extracted with some degree of ambiguity, e.g. the suffix -s may be in Spanish a plural (“las compras -the purchases-”) or it can be the second person of a verb (“tu compras -you buy-”). From a probabilistic perspective, predictions regarding the effects of surface and root frequencies warrant reinterpretation, given that both variables provide information about the syntagmatic combinatorial properties of morphological structures inside the words (roots and suffixes), and not about the access and representation of the whole word. Against the traditional perspective, the surface frequency could be interpreted (Baayen et al., 2007) as an indicator of morphological structure and not as the information of the whole word familiarity. In particular, the surface frequency of inflectional plurals would indicate the frequency with which a root combines with the suffix –s to form a plural. This interpretation, therefore, predicts surface frequency effects for plural dominant over its corresponding non-dominant forms as a sign of morphological computation of number. However, the results of previous experiments in Spanish (Dominguez et al. 1999) do not align well with this prediction. The absence of a dominant frequency effect for plural dominant items supports better the prediction of the classical point of view in which singulars are not decomposed whereas plurals are accessed through their corresponding singular form.

From our point of view, these apparently contradictory results and interpretations could be partially reconciled by adopting a particular paradigm, such as is the singular/plural of the same stem, to achieve a well-controlled situation, manipulating only the surface frequency of each word. However, beyond controlling for spurious variables that might influence morphological processing experiment outcomes, investigating the age and experience of readers with simple and compound words might be crucial.

### Age and Inflectional Morphology

Reifergerste et al. (2017) proposed two new conditions that determine the recognition of morphologically complex words: the experience of readers, and the morphological complexity of the language (see also Clashen & Reifergerste, 2017; Lorenz et al., 2019, and Reifegerste et al., 2019 for morphological differences due to age). Unlike adults, young German readers (i.e. pregraduate students) do not exhibit the typical disparity in lexical decision times between singular dominant-singulars (e.g. cometa) and plural dominant-singulars (e.g. diente). The lexical decision times of young readers for singulars, as for plurals, depend on the cumulative frequency of singular and plural forms but will be shorter than those of plurals, because, in addition to being affected by the base frequency, they have to be segmented in stem and affix. In view of these results, the authors defended an extended morphological analysis for all lexical items (Taft, 2004; Taft & Forster, 1975) in young readers.

Similar to Reifergerste et al. (2017), age-related differences were observed in our study of gender processing in Spanish. Santos et al. (2022) compared 8 and 11-year-old children with adults. The data revealed a distinction between feminine and masculine forms of masculine-dominant pairs, whereas for feminine-dominant pairs, no differences between masculine and feminine forms emerged. On the contrary, the adult processing of gender in Spanish seems to be carried out through a direct route of processing, given that the recognition of feminine and masculine words was influenced only by its surface frequency and not by the base frequency (Butterworth, 1983; Mannelis & Tharp, 1977; Rueckl et al., 1997). In terms of probabilistic interpretations as Baayen et al. (2007) account, however, the extended effect of surface frequency indicates not only the familiarity of the whole word but also the familiarity of the combination of root and affixes in compound words. However, the mechanisms underpinning children’s access differ for masculine and feminine genders, pointing towards a classical dual-access explanation. That is, a route of segmentation would be used for feminine words, better predicted by the base frequency, whereas the direct route would operate with masculine words, better predicted by surface frequency. Differences with adults could be determined by the *paradigmatic* ambiguity of gender in Spanish. Although the more frequent inflections are “–a”, for feminine words, and “–o”, for masculine words, there are some exceptions to this rule and many other endings of words marking gender (see Teschner & Russel, 1984). This ambiguity is also present in Spanish plurals, as it was remarked before, given that the final -s could be indicating not a plural but also the second person of verbs (e.g. las compras vs. tú compras [the purchases vs. you buy).

Given the differences due to age in a morphologically complex paradigm, as is plural in German, and also due to previous data obtained in Spanish gender processing, an additional goal of this study is to know the impact of age over the Spanish morphological processing of number. We intended to contrast Spanish children of Third grade (aged 8–9), Sixth grade (aged 11–12) and Adults, in a lexical decision task, testing the hypothesis that less experience in reading will promote the extended influence of base frequency even in a language with a unique suffix of plural. We might think that the simplicity of the plural suffix makes the question posed by our research irrelevant: since identifying the plural is as simple as recognizing a letter at the end of the word, why not simply do this? However, this process may be slower than direct access to a representation of the complete plural word in memory, along with its associated meaning. The morphological process requires segmenting the suffix, identifying the stem of the word, and accessing lexical memory through the singular form. Therefore, direct access could be faster and more efficient, but it requires prior storage of the plural form, which can only be achieved through repeated exposure to that plural form, or in other words, experience. This is why it is so important to measure lexical access at different ages to observe the effect that experience can have on the use of the direct or indirect morphological route.

By using pairs of singular/plural words with the same stem (e.g., diente/dientes) that differ only in surface frequency, we control certain intervening variables in visual word recognition, such as family size or orthographic similarity. This allows us to focus directly on the difference between singulars and plurals when these forms are frequent or infrequent compared to their corresponding counterpart form.

It is expected that the results of Dominguez et al. (1999) will be replicated in the group of adult participants: the dominant singular will produce shorter times than its corresponding plural. However, the dominant plural will produce the same reaction times as its corresponding singular. Any other result should be interpreted according to the parameters of probabilistic models such as that of Baayen et al., (2007, 2011), especially if a dominance effect is obtained in the plural form over the singular. With respect to the children’s groups, it would be possible to find no influence of the dominant frequency variable, like in German. However, unlike German, Spanish simplicity of plural, (i.e. -s) could facilitates the recognition of the singular and the plural forms, receiving, in this case, the influence of the frequency of each form.

## Method

### Stimuli and Design

The experiment includes two within-participant variables: number (singular and plural) and dominance (dominant and not dominant), as well as a between-participants variable: group, (third grade, aged 8–9; sixth grade, aged 11–12; and university adults).

Sixty pairs of singular-plural words were selected. Thirty of these pairs were singular dominant “cometa/cometas” [kite/kites], and the other thirty were plural dominant “diente/dientes” [tooth/teeth]. Each participant saw fifteen singular words and fifteen plural words from singular dominant pairs, and fifteen singular words and fifteen plural words from plural dominant pairs. All the nouns and adjectives were extracted from the “Diccionario de Frecuencias del Castellano Escrito en niños de 6 a 12 años –Frequency dictionary of written Castilian for 6 to12 year old children” (Martinez-Martin & Garcia Perez, 2004), a specific dictionary of lexical frequency for children. The construction of this dictionary started with the selection of a sample of children from first to sixth grade in elementary school. The frequency of the words was obtained from the books read by these children over the academic year, including the textbooks. Consequently, it is a measure of the written frequency of words similar to that of adult frequency dictionaries.

The participants also encountered a total of sixty pseudowords, with half being plurals ending in –s, and the other half singulars, typically ending in –a or –o, reflecting the common gender endings in Spanish. The pseudowords were formed changing one letter from an existing word in the initial, middle or final part (except for the last letter, which was never changed).

All categories of stimulus were matched in lexical frequency (see Table 1). By design, plural forms always had one more letter than singular forms, due to the addition of the suffix -s to denote plurality in Spanish. Therefore, the difference between singular and plurals is necessarily confounded with length, but it must not compromise the main interest of this study on the interaction of the dominance of frequency and the number (see stimuli sets in the Appendix).

**Table 1:** Average values of frequency and length in each experimental condition

	Singular		Plural	
	Frequency	Length	Frequency	Length
Singular Dominant	560.02	5.96	186.95	7.13
Plural Dominant	188.7	6.26	566.7	7.56

Since the same stimuli were presented also to the adult group, a valid question might be whether the dominance relationships in frequency, as established using the children’s dictionary, would still hold when measured using a general adult dictionary. It can be seen in the Appendix the frequencies from the children’s dictionary (Frequency column) and those from a general adult frequency dictionary (NIM column) of Guasch et al. (2004). The dominance relationships established with the frequencies from the children’s dictionary were preserved for all singular-plural pairs in the adult dictionary. The correlation between both measures was significant (r^2^ = 0.744, *p* < 0.0001), thereby confirming that the stimuli selected for children were also appropriate for the adult group.

### Participants

The experiment involved two groups of children: fifty third-graders aged 8–9 years (29 boys, 21 girls), and fifty-two sixth-graders aged 11–12 years. In third grade, students are acquiring fundamental skills in phonological decoding and reading fluency. By sixth grade, it is expected that students have developed a broader vocabulary based on reading more sophisticated texts. Comparing these two grade levels allows for monitoring progress to observe any changes that may occur in the use of the direct and indirect routes of morphological processing.

None of the students were enrolled in special education or reading recovery programs. Their academic performance was around the average for their respective grades. All participants had normal or corrected-to-normal vision and were Spanish native speakers. They voluntarily took part in the experiment. Parents were informed about the aim and procedures of the experiment via a letter, and they signed the corresponding consent forms.

The two participating schools are located in downtown Santa Cruz and serve neighborhoods with a diverse socioeconomic mix. Most families in these schools are of high to middle socioeconomic status, with parents having at least a high school or university level education.

The group of adult readers was composed of 72 participants between 18 and 30 years old (63 women and 9 men), undergraduate students from the Speech and Therapy degree of the University of La Laguna. All of them were informed of the procedure and general goal of the experiment and participated voluntarily. They received academic credits for their participation.

### Procedure

The participants were individually tested in a soundproof and isolated room either at their respective schools, or in the Faculty Laboratory for undergraduates. All participants performed a lexical decision task, a common method for studying lexical access (Meyer & Schvaneveldt, 1971, Ehri & Wilce, 1983; Perfetti & Hogaboam, 1975). The participants were instructed to respond as quickly and accurately as possible, identifying whether or not the presented stimulus was a word or a pseudoword. They should press the “SI” button with the index or heart finger of their right hand (labeled on the “L” key of the keyboard) when a word was presented, and press “NO” with their left index or heart finger when a nonword was presented (labeled on the “S” key of the keyboard). Stimuli were administered with the E-prime 2.0 program (Schneider et al., 2002) which also recorded the reaction times and errors. The stimuli were preceded by an asterisk as a fixation point during 1000 ms and appeared in the center of the screen in white letters on a black background, remaining there until the response of the participant. Latencies from the stimulus appearance to the participant response, and errors, were recorded.

The experimental stimuli started to be delivered after the children completed 10 items of training and once the researcher was sure the participant had understood the instructions. The stimuli were randomized across participants and presented in the center of the screen with a 70-Hz refresh rate. The letters, presented in lower-case case Courier 16 font, appeared as white characters on a black background. Each character covered approximately 0.38° of visual angle from a distance of 60 cm.

## Results

Table 2 shows mean reaction times (RTs) calculated once errors were removed as well as mean percentage of accuracy for each experimental condition in each group of age. As for the trimming RTs data, response errors were first removed from the analysis (*n* = 447, 4% of trials). Further, trials with RTs faster than 0.01-quantile (385 ms, *n* = 100) or slower than 0.98-quantile (2617 ms, *n* = 204) were considered as absolute outliers and excluded from the RTs analysis (0.1%). Trials with RTs that deviated more than 2.5 SDs from the participant’s mean latency (relative outlier trials) were also discarded (*n* = 264, 3%), leaving 9605 data points for main data analysis.

**Table 2:** Mean reaction times, square error and percentage of accuracy obtained for the experimental conditions of the experiment

	Target word			
	Singular Dominant		Plural Dominant	
	Singular	Plural	Singular	Plural
Groups	Paseo (walk)	Paseos (walks)	Diente (tooth)	Dientes (Teeth)
Third				
Mean RT	1271	1346	1315	1332
SE	20.38	21.48	21.78	21.84
Accuracy (%)	96.5	94.6	92.3	95.2
Sixth				
Mean RT	1100	1185	1144	1172
SE	16.56	18.40	17.90	18.65
Accuracy (%)	98.1	96.3	98.3	95.6
Adults				
Mean RT	592	638	622	630
SE	4.58	5.92	5.07	5.56
Accuracy (%)	95.9	95.9	93.7	96.1

The data were analyzed with generalized linear mixed-effects (gLME) models (Baayen et al., 2008) using function glmer of the lme4 package v. 1.1–27 (Bates et al., 2015) in R version 4.1.0 (R Core Team, 2018). Specifically, Gamma family and identity link were used in models fitted to continuous data, and the Binomial family and logit link to binomial variables. Reaction times and accuracy data were predicted by Dominance (singular vs. plural dominance), Number (singular vs. plural) and Groups (the third- and sixth-grade children and adults). All categorical variables are sum coded (2-level predictor Dominance and Number, − 0.5, 0.5; 3-level predictor Groups, − 0.5, 0.5, 1), such that the effect estimates would be evaluated at the grand average across all predictors and thus can be interpreted as simple main effects. In all models, we applied a model trimming approach starting with a maximal model including random intercepts for participants and items, by-participants random slopes for Dominance and Number and their interactions and by-item random slopes for Groups. Subsequently, if a model failed to converge, we followed Barr (2013) and Barr et al. (2013) suggestions simplifying the maximal model, by removing correlations between random factors until nonsingular convergence was achieved. Resulting models were compared to the full model (with maximal random structure) using the chi-square difference test and Bayesian’s information criterion (BIC), for which a lower value indicates better model fit (Kline, 2011). We called function *car:Anova*() from the *car* package to test the significance of effects and calculate *p-*value. Using chi-square instead of *F* value avoids issues in estimating the dominator degree of freedom in unbalanced design and is analogous to treating t-distribution as a z-distribution for the individual coefficients (Alday et al., 2017). Post-hoc comparisons were performed using the glht function in the multcomp package (Hothorn et al., 2015), with free method in order to take the correlation of the model parameters into account. The data and scripts to replicate the analyses are available at the Open Science Framework at https://osf.io/ka7g9/?view_only=787c16b7a05e4272a7ddf38828629b8d.

The analyses (Table 3) showed a significant effect of Number factor indicating that singular words received shorter reaction times than plural words and the Dominance factor indicating that dominant gender produced shorter lexical decision. Also, the factor Group produced significant differences given the group Third produces longer reaction times the group Sixth and the last produces slower responses than the adult group. Both factors interacted significantly, indicating a greater difference between singular and plural when the singular is dominant (69 ms) than when the plural is dominant (18 ms). This influence of number dominance on the number variable was confirmed in the triple significant interaction across groups. The Table 4 show the follow up comparisons between plural and singular for plural dominant pairs revealing a non-significant difference of 17 ms in the group Third, a non-significant difference of 28 ms in the group Sixth and also a non-significant difference of 8 ms in the adults group. On the contrary, for the plural dominant pairs the 75 ms difference between the plural and the singular forms in the group Third resulted in a statistically significant effect, as well as the 85 ms difference for group Sixth and the 46 ms difference for the group Adult.

**Table 3:** Chi-square values and probability for each of the factors of the experiment and interactions

Analysis of RT data across groups	
Factors	
Dominance	χ^2^(1) = 65.33, *p* < .0001***
Number	χ^2^(1) = 19.87, *p* < .0001***
Group (Sixth grade)	χ^2^(2) = 70,931.78, *p* < .0001***
Dominance × Number	χ^2^(1) = 121.42, *p* < .0001***
Dominance × Group	χ^2^(1) = 17.29, *p* < .0001***
Number × Group	χ^2^(2) = 11.54, *p* = .003**
Number × Dominance × Group	χ^2^(2) = 104.83, *p* < .0001***

**Table 4:** Pos-hoc comparisons to disentalgle the triple interaction between the three main factores

Follow-up comparisons									
Plural and singular contrasts across dominance in each group									
Group	Contrasts	Plural dominant				Singular dominant			
		Estimate	*SE*	z	*p*	Estimate	*SE*	z	*p*
Third	Plural versus Singular	13.17	6.26	2.104	0.106	83.48	7.05	11.845	< .0001
Sixth	Plural versus Singular	12.29	6.88	1.786	0.148	73.48	8.89	8.262	< .0001
Adult	Plural versus Singular	− 3.49	6.86	-0.510	0.610	38.11	7.77	4.906	< .0001

Regarding the analysis of accuracy (see Table 5), the singular forms produced a significantly higher number of correct lexical decisions than the plural forms. The group of Sixth was more exact in his/her responses than the group of third and unexpectedly, also than the group of Adults.

**Table 5:** Analysis of accuracy data across groups

Factors	
Dominance	χ^2^(1) = 1.05, *p* = .3
Number	χ^2^(1) = 4.52, *p* = .03*
Group (Sixth grade)	χ^2^(2) = 6.89, *p* = .03*
Dominance × Number	χ^2^(1) = 1.91, *p* = .17
Dominance × Group	χ^2^(1) = 0.86, *p* = .65
Number × Group	χ^2^(2) = 6.16, *p* = .04*
Number × Dominance × Group	χ^2^(2) = 1.48, *p* = .48

The interaction between number and group showed a higher number of right responses for singular than for the plural words in the group Third and also in the group of Adults, but on the contrary, in the group of Sixth, the highest correct responses corresponded to the plural words. This difference was confirmed in the post-hoc analyses as may be seen at Table 6.

**Table 6:** Follow-up comparisons for the differences between plural and singular in the three groups analysed

Follow-up comparisons					
Groups × Number					
Group	Contrasts	Estimate	SE	z	*p*
Third	Plural versus Singular	− 0.14	0.36	− 0.38	.9
Sixth	Plural versus Singular	− 1.18	0.39	− 3.05	.007
Adult	Plural versus Singular	− 0.21	0.28	− 0.75	.9

**Fig. 1:**
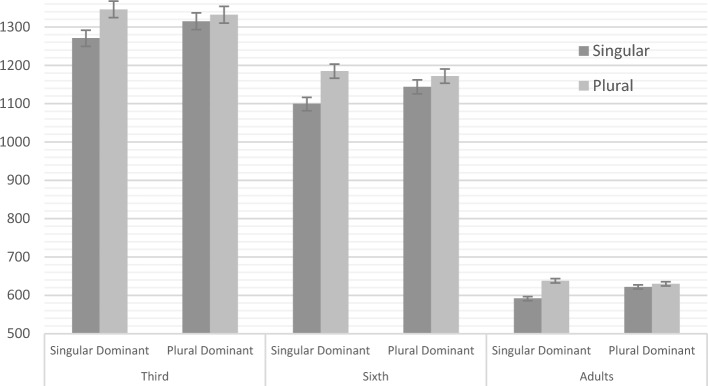
Evolution of the lexical decision times (and standard error) on the words of singular dominant pairs and plural dominant pairs from the third grade (left) and the sixth grade (center) to the adult’s group (right). Note the superficial frequency determine the difference between singular and plural in the singular-dominant pairs across ages but not in the plural-dominant pairs

In summary, the groups of children of 8 and 11 years old, and adults show a similar pattern of results (see Fig. 1) with an interaction between dominance and number that support a different processing for singular and plural words. These experimental results replicate a previous study (Dominguez et al., 1999) carried out only with adults.

## Discussion

Two groups of children from the Third and Sixth grades of Elementary School and a group of Adults were tested reading plural and singular words of the same stem. The participants were asked to make a lexical decision of singular and plural words from singular dominant pairs and plural dominant pairs. Dominance was defined by a higher frequency of the singular or plural words of the same lexeme: “*dientes-diente*” [tooth-teeth] is a plural-dominant pair and “*paseo-paseos*” [walk-walks] is a singular-dominant pair.

Lexical decisions of children were two times slower than those of adults (see Fig. 1), but the pattern of children’s reaction times behaved as in the adult group and also as in previous research (Dominguez et al., 1999): plurals of plural-dominant pairs produced similar lexical decision times as singulars of plural-dominant pairs, providing evidence that plural recognition could be dependent on the accumulative frequency of the lexeme. On the contrary, the singular of singular-dominant pairs speeds the responses due to its own high frequency, whereas plurals of these pairs are probably recognized through previous recognition of the stem (i.e. the singular form), slowing down its reaction time.

The results support a system of recognition based on the classical dual procedure: a direct route to the whole word representation in memory which is affected by the frequency of items and an indirect subsidiary route that separates the lexeme and the suffix to accede to the morphological information of number. This indirect route, in the case of number processing, is only possible for plurals, because there is no singular suffix in Spanish. The recognition times of singulars do not differ of plurals when they are more unfamiliar than their corresponding plural but differs when the singular is more frequent than plural. The race between the direct and the indirect routes starts at once but the final result is determined, in the case of singulars, by their superficial frequency, and in the case of plurals, by the process of segmentation and the frequency of the base morpheme (Schreuder & Baayen, 1995). Any other alternative explanation like those based on the interpretation of surface frequency as a sign of morphological treatment of the word (Baayen et al., 2007, 2011) does not seem plausible, given the absence of an advantage of plural dominant items over the singular nondominant forms.

No differences were found between the results obtained by the children and those of adults in the experiment. The age does not determine a change in the procedure used to recognize plural of words by young beginner readers and young adult skilled Spanish readers. This is a different result than in German, language in which the adults recognize singular and plural differently than do young people (Reifergerste et al., 2017). Young German readers recognize singulars and plurals through the stem, which accumulates the frequency of both, the singular and the plural forms, and as a result, singulars and plurals of the same stem received similar lexical decision times irrespectively of the dominance. The decisive factor in explaining results in the young German readers, offered by the authors, was an interaction between the accumulated experience with the language and the high number of suffixes of plurals. The rich morphology of the German language compels young readers to give priority to the stem, probably by identifying it as a significant segment which, in combination with a few suffixes allows to obtain the meaning of thousands of morphologically complex words found in the first time. However, the Spanish inflectional system has only one suffix for the plural: “–s”. When children and adults encountering a plural, they are likely to segment it to access the base form, the singular, regardless of the plural’s frequency. In view of the results, the age of participants does not seem to be a sufficient condition to explain by itself the prevalence of cumulative frequency of singulars and plurals over the surface frequency found by Reifergerste et al. (2017), because even when the age of the participants of our study was notably lower, our results differ substantially of those. Consequently, the simplicity of Spanish, with a unique plural ending, facilitates the identification of the morphological structure of the word in the case of plurals but the frequency of the plural is not taken in account when recognizing the singular form. Additionally, the ambiguity of the orthographic mark of the plural, which can also function as a verbal form, does not influence a different pattern of morphological structure learning. Note that this ambiguity does not exist when the words appear inside sentences. The word “compras –purchases-” can be a noun and then it will be preceded by an article, i.e. “las compras –the purchases-”, or it can be a verb, and then it will be preceded by a second person pronoun, i.e. “tú compras –you buy-”, or by the omission of the same, also valid in Spanish. In any case, when the word “compras” appears alone, it does not matter whether the final -s corresponds to a noun or a verb, as morphological segmentation is applicable in both cases. Therefore, it would produce stem-dependent reaction times as well.

An additional issue raised in light of these results is the flexibility and adaptability of the morphological system of processing. One interesting point arising from these results is the efficiency of a reading system that requires two different procedures—direct access and the use of segmentation rules—to reach the meaning of words. The choice between these procedures depends on several factors, as suggested by the probabilistic account of morphological processing (Baayen et al., 2007, 2011). These factors include the frequency, age, number of suffixes in the language, productivity and type of suffixes (inflections or derivations), and regularity among others. Our point of view is that most of these determinant variables may be reduced to a common factor: the experience of readers with the roots and the suffixes forming different words. The familiarity of words to the beginner readers is reduced due to their lack of experience. Morphological segmentation allows them to grasp the meaning of unfamiliar complex words through the more frequent and likely known morphemes that make up these words, as opposed to recognizing the entire word. Age of acquisition of base words is, in fact, earlier than the age of complex words (Davies et al., 2016), perhaps because these base words take part in many other words, and readers are more familiar with these configurations. Adult readers, on the contrary, have many more representations of whole words by repeated exposure, increasing the capacity of the direct access route. Therefore, age and frequency could be consolidated into a single factor, namely, the number of exposures to the words in the language.

Conversely, morphological productivity and the number of suffixes in a specific paradigm, such as gender or number, enable speakers and/or readers to understand the meaning of a word via the stem and affixes, even if they do not recognize the entire word. Thus, we posit that the language system toggles between direct and indirect procedures through a statistical exploration of the language. This view is in consonance with the probabilistic model of Baayen (2007, 2011), which points to paradigmatic and syntagmatic factors influencing the access and representation of morphologically complex words.

According to the results presented here, the plurals are formed in such a simple way in Spanish, and some other languages, that the use of both procedures, direct and indirect, does not change throughout the life. Another factor simplifying the learning of plurals in Spanish is that the ‘-s’ of plural nouns repeats in adjectives, determinants, pronouns, and other functional elements that agree with the noun to conform to the sentence’s syntactic structure, such as ‘niños guapos’, ‘los niños’, ‘estos niños’, etc. ([handsome boys], [the boys], [these boys]). Therefore, the application of rules is useful not only at the lexical level but also at the syntactic level. The Dual Route Race Model (Schreuder & Baayen, 1995) remains the best option to accommodate the access and representation of number along the life in Spanish.

Some conclusions could be derived of our results:

- The plural form of each noun or adjective is identified through the singular form, which is directly recognized in the lexical memory
- These two forms of recognition do not vary from childhood to adulthood
- The developmental differences observed in other languages, such as German, seem to be dictated by the simple structure of plurals in Spanish
- The identification of a suffix -s at the end of the word and some syntactic agreement cues in the sentence allow the child to discriminate the plural from the beginning of their reading experience.

A limitation of this study could be the exclusive use of isolated words. Future developmental research could investigate whether the advantage of dominant singular frequency words disappears when they are inserted into a sentence context with determiners that agree with them in number.

## Appendix

This table shows the singular and plural words used in the experiments.

NOL = Number of letters; NIM = Adult frequency from NIM dictionary (Guasch et al., 2013).

**Table:**

	Frequency	NIM	NOL	Plural	Frequency	NIM	NOL
Singular dominant							
*List 1*							
lugar	1568.38	367.72	5	lugares	267.84	63.774	7
nombre	1466.15	271.083	6	nombres	589.11	79.939	7
cuento	382.64	48.852	6	cuentos	187.28	23.271	7
regalo	251.68	29.133	6	regalos	132.61	14.211	7
domingo	228.65	62.886	7	domingos	86.31	22.383	8
banco	192.72	46.72	5	bancos	47.3	36.594	6
camión	165.12	21.317	6	camiones	35.56	15.455	8
cero	131.34	19.363	4	ceros	65.68	1.954	5
metal	84.84	18.297	5	metales	30.47	9.415	7
retrato	68.9	29.489	7	retratos	11.55	8.527	8
cometa	65	7.461	6	cometas	30.66	3.02	7
cómic	57.31	0.711	5	cómics	12.89	0.711	6
concierto	53.24	12.435	9	conciertos	18.81	8.349	10
chándal	50.25	1.244	7	chándales	3.13	0.178	8
puzzle	33.52	2.842	6	puzzles	3.57	0.355	7
	319.98	62.63	6		101.51	19.20	7.2
*List 2*							
número	1477.86	237.686	6	números	775.82	25.936	7
texto	488.24	66.971	5	textos	53.86	30.377	6
sueño	358.95	131.456	5	sueños	141.81	50.273	6
círculo	248.7	26.291	7	círculos	62.98	14.034	8
tesoro	195.09	15.455	6	tesoros	37.54	7.461	7
paseo	181.52	25.758	5	paseos	28.25	11.547	6
tejado	146.36	11.014	6	tejados	44.64	11.192	7
premio	116.97	33.752	6	premios	30.75	14.389	7
milagro	71.26	35.884	7	milagros	30.34	14.034	8
truco	68.34	9.06	5	trucos	33.2	6.928	6
saludo	61.22	13.501	6	saludos	15.54	3.553	7
taxi	53.99	22.383	4	taxis	3.75	4.619	5
televisor	53.18	20.429	9	televisores	6.1	4.619	11
molino	48.16	8.527	6	molinos	11.94	2.487	7
mantel	30.7	5.862	6	manteles	4.93	1.954	8
	240.03	44.26	5.93		85.43	13.56	7.07
Plural dominant							
*List 1*							
ojos	1950.8	482.833	4	ojo	300.9	70.879	3
animales	1370.28	108.54	8	animal	583.3	72.656	6
instrumentos	337.15	20.074	12	instrumento	181.57	28.245	11
peces	263.51	16.698	5	pez	145.04	15.455	3
huesos	216.69	35.173	6	hueso	96.5	15.1	5
caramelos	190.51	4.974	9	caramelo	48.75	2.842	8
centímetros	158.42	30.91	11	centímetro	35.92	7.461	10
grados	123.69	53.65	6	grado	58.64	29.84	5
arbustos	86.08	4.086	8	arbusto	31.81	1.421	7
rotuladores	69.1	0.711	11	rotulador	31.12	0.533	9
cuernos	68.83	10.836	7	cuerno	38.74	6.395	6
aplausos	54.3	12.257	8	aplauso	13.96	6.04	7
bombones	51.03	3.731	8	bombón	16.17	1.776	6
patines	50.14	1.421	7	patín	6.95	0.711	5
pétalos	32.08	6.217	7	pétalo	5.38	1.421	6
	334.84	52.80	7.8		106.31	17.38	6.46
*List 2*							
Plural							
años	1434.7	1168.18	4	año	704.45	342.14	3
dientes	475.63	67.504	7	diente	59.63	6.928	6
huevos	329.09	29.311	6	huevo	191.58	20.251	5
zapatos	236.76	44.233	7	zapato	66.46	12.968	6
labios	191.28	109.428	7	labio	28.9	7.994	6
apóstoles	174.81	3.908	9	apóstol	15.43	3.731	7
juguetes	140.71	7.106	8	juguete	58.92	5.329	7
insectos	115.24	14.389	8	insecto	27.08	5.507	7
sellos	72.03	6.217	6	sello	28.49	4.974	5
cromos	68.94	3.02	6	cromo	12.89	1.954	5
guantes	58.15	9.948	7	guante	14.55	8.349	6
cereales	52.53	5.685	8	cereal	3.76	1.421	6
calzoncillos	50.51	8.172	12	calzoncillo	4.77	0.888	11
reptiles	47.16	5.329	8	reptil	13.56	3.198	6
talones	30.33	7.816	7	talón	5.23	3.731	5
	231.85	99.34	7.33		82.38	28.62	6.06

**Table:**

Pseudowords			
Singular Pseudowords	Number of letters	Plural Pseudowords	Number of letters
babo	4	descos	6
piobla	6	parros	6
despeto	7	medres	6
teso	4	sories	6
gablante	8	tenostos	8
repla	5	últamos	7
lamello	7	pideos	6
planja	6	delatos	7
guetero	7	serpiuntes	10
potanco	8	hulados	7
cutado	6	chalos	6
maire	5	chimoneas	9
cala	4	vajeos	6
ponchu	6	cervecarías	11
infoliz	7	celabros	8
zita	4	acoros	6
pelato	6	brochizos	9
cantesto	8	crominales	10
asmo	4	guscazos	8
blonca	6	posisos	7
bafetón	7	ratrasos	9
azécar	6	tateajes	8
corbuta	7	teclos	6
depirte	7	zarrones	8
ostrella	8	licros	6
lunu	4	colmallos	9
llavo	5	cenozas	7
refaño	6	ostrenos	8
sapa	5	sóptimos	8
lluvoa	6	infocciones	11
	5.97		7.63
